# Perception and reaction of Nanyang Technological University (NTU) researchers to different forms of research integrity education modality

**DOI:** 10.1186/s12910-022-00824-6

**Published:** 2022-08-24

**Authors:** Jolene Y. L. Chua, Celine S. L. Lee, Kwee P. Yeo, Yusuf Ali, Chin L. Lim

**Affiliations:** 1 Lee Kong Chian School of Medicine, Nanyang Technological University, Singapore, Singapore; 2 School of Physical and Mathematical Sciences, Nanyang Technological University, Singapore, Singapore

**Keywords:** Research integrity, Research integrity education, Research ethics, Scientific misconduct

## Abstract

**Background:**

Research and academic institutions use various delivery channels to deliver Research Integrity (RI) education in their communities. Yet there is no consensus on the best delivery method and the effectiveness of these channels in inculcating a positive RI culture varies across institutions. Hence, this study aimed to understand the preferences of the research community in Nanyang Technological University (NTU), Singapore.

**Methods:**

An online survey was conducted on NTU research community to understand their experience with, and preference for each RI education mode offered in NTU. The RI education modes surveyed in the general ranking question are Data Management Plan (DMP) workshops, Epigeum e-Learning, Compass e-newsletter (email), and NTU policy on Research Integrity and Responsible Conduct of Research. There were 242 responses, comprising 50% research students, 32.2% research staff and 17.8% faculty members. Non-parametric statistical techniques were used to analyse preferences across different RI education modes and within sub-groups (i.e., fields, age, native language, roles in research community).

**Results:**

More than 92% of respondents subscribed to the importance of RI education, but with different preferences for education modes. With respect to RI education in NTU, Compass e-newsletters were ranked the lowest (*p* < 0.05). Most felt that they were too wordy and unengaging, making it difficult to absorb information. Similarly, Epigeum e-Learning (*p* < 0.05) and ‘policy’ (*p* < 0.05) were found to be too lengthy in presentation. The compulsory NTU RI education modes (Epigeum e-learning and ‘policy’) enjoyed higher participation rates of 70–80% compared with 32–37% for the self-regulated modes (DMP workshop and e-newsletter). This suggests that regulatory mechanisms are still necessary to promote participation in RI education, and thus, core RI education content should be made compulsory in research/academic institutions. Although Epigeum is a compulsory course, some may not have participated in the programme due to technical issues or they might have forgotten to participate in the programme within the permissible timeframe. For all four RI education modes in NTU, the lack of awareness was among the top cited reasons for not participating.

**Conclusions:**

Most NTU researchers perceived RI education positively although they may have reservations for some approaches. Conversely, e-Learning is favored over all the other modes except for the mode of Policy. Findings from this study are useful for improving the design of RI education strategies to be more appealing to the research community by enhancing user experience in terms of user-friendliness, relevance to specialisation, providing concise information and better presentation of materials For institutions with similar modes of RI education as NTU, these results may be relevant in improving participation rates and presentation of RI education modes, such as the use of infographics and more concise information.

**Supplementary Information:**

The online version contains supplementary material available at 10.1186/s12910-022-00824-6.

## Background

Research integrity (RI) forms the foundation of good research practice, and its preservation depends upon individual behaviours as well as the overall research climate and attention given by the leadership of academic and research institutions [1]. In spite of strict governance systems enacted by scientific communities, academic institutions and publishers to uphold RI, unethical or questionable research behaviours of varying magnitudes persists [2]. These practices range from cases of scientific misconduct, such as blatant fabrication of results or subjecting research participants to unapproved and harmful procedures, to more questionable behaviours, such as improper authorship assignment [3]. Regardless of severity, these practices not only undermine progress in science, but also weaken the trust placed upon scientists and research institutions by public administrators and members of the public at large.

Among the variety of interventions available to promote RI, training programs are most prevalent as they are mandated by several funding bodies such as the National Institute of Health (NIH) and the National Science Foundation (NSF) in the United States of America. Numerous modes of delivery and pedagogy are employed in RI training programs, from self-paced online courses to face-to-face sessions or a hybrid model of the two delivery modes. In general, the choice of pedagogy and communication for RI training programs mirrors the institutional requirements governing responsible research practices [4]. For example, NIH mandates at least eight contact hours, while NSF provides no specific guidance or requirements for the structure or format of the training program [5]. There are numerous guidelines on RI globally, which is beyond the scope of this study to cite and address. In Singapore, the National Research Foundation encourages host institutions to provide training on responsible conduct of research and these RI training programs are executed by the respective funding agencies and institutions with their own set of guidelines and requirements. Most Institutional Review Boards (IRB) also require researchers to have certifications related to RI and good research practice when submitting proposals to the board (e.g., Collaborative Institutional Training Initiative, or CITI) [6]. In NTU, the IRB requires all researchers to complete approximately 8 h of online training and to have CITI certification when applying for IRB approval.

Aside from funding institutions and the IRB, the importance of buy-in from members of the research community cannot be understated [7]. Aspiring researchers need to embrace RI education as something more than a compulsory academic module or institutional key performance indicators. Involving the faculty in RI education is a crucial step in achieving this. Their expertise adds legitimacy and their experiences provide relatable context to the principles [4]. Having a systematic and structured approach to RI education also helps to set an explicit standard for upholding RI within an institution.

Despite the consensus on the importance of RI training, there are mixed findings regarding the effectiveness of pedagogy and delivery modes. This inconsistency in findings can be attributed to difficulty in evaluating the effectiveness of the respective RI training programs. To circumvent the limitations, self-reported outcomes, or surrogate measures, such as reaction to training interventions and changes in RI-related attitudes and knowledge, are used instead to indirectly assess the effectiveness of RI training programs. While some meta-analytic studies [8–10] demonstrated that RI training had small to moderate impact on individual behaviour, these results should be interpreted with caution because their external validity is limited by the lack of systematic and rigorous evaluation of ethics instruction [9]. One study used a single index across courses in the United States and found low to moderate effects of RI training on ethical decision making among researchers [11]. In a Cochrane Review, Marušić et al. [12] concluded that the efficacy of training in responsible conduct of research remains uncertain due to the poor quality of evidence. Hence, as evaluation of RI training is complicated, it is not known which educational approaches work well.

However, there are aspects of RI that can be taught and learned, especially if they relate more to convention than moral attributes, as the latter, while important, is more challenging to teach and assess [13]. Examples of convention-related attributes include authorship assignment and publication requirements. Authorship assignment follows a well-established set of guidelines which are supported by consensus in the scientific community. Without sufficient knowledge of these guidelines, researchers are more likely to fall prey to common pitfalls of authorship assignment, such as honorary authorship. Hence, RI education is valuable for reducing the likelihood of misconducts that commonly result from ignorance and uncertainty over conventional attributes of RI rather than immoral intentions [14].

Beyond training programs, many institutions (e.g., MIT, Cambridge University) have also designed policies and support mechanisms to promote and govern RI [13]. In Singapore, the NRF requires the host institution to have a research integrity policy. Policies or codes of conduct lay the foundation for responsible conduct of research and outline procedures for managing allegations. Researchers are expected to be familiar with, and be guided by these policies as part of their routine research practices [15]. A more direct and thorough intervention to govern RI is through the audit process, or compliance monitoring. Audits are common for instance (but not exclusively) in clinical trials to safeguard patient’s well being as well as to ensure data integrity and reliability [16]. Auditing the spectrum of practices, from research protocols to publications, allows questionable practices to be highlighted and research misconduct to be mitigated. If the misconduct arises from the lack of knowledge, immediate and targeted instruction can be provided [17]. Other forms of intervention or instruction include mentorship and independent peer reviews.

While majority of the literature remain focused on the effectiveness of RI education, few empirical studies have provided a macro view by comparing the preferences of members of the research community for different RI education channels. Effectiveness is about measuring researchers’ awareness and compliance of good RI practices, while preference refers to how researchers perceive the different RI education approaches. Rather than focusing on effectiveness, which is difficult to measure and evaluate, this study sought to gain clarity on researchers’ preference of RI education channels instead. Given the variety of RI education channels, it is possible that researchers feel overwhelmed, and the multifaceted strategy to promote RI may produce increasingly marginal outcomes. It would thus be advantageous to prioritise channels for RI education that are preferred and minimise investing in education channels that are less appealing to researchers.

Educational research has established that “spaced review and practice” (learning that occurs over an extended period, with frequent opportunities for practice and feedback) leads to greater retention of information and longer-term behaviour change than learning that occurs intensively during a brief period” [15]. In the context of RI education, it is worthwhile to deliberate how the different approaches can be spaced out based on preferences to maintain interest over time. Sefcik et al. [2] also noted the importance of timing in engaging researchers. Some modes of instructions should be flexible such that researchers are exposed to the content at an opportunistic time. Therefore, comparing preferences for different approaches provide valuable information that will inform prioritisation and even classification based on essential and elective courses. Such data would be important in guiding the strategy for designing RI education to achieve optimal outcomes.

NTU employs a variety of modes/channels to reinforce a culture of honesty, transparency, accountability and ultimately a robust sense of RI. These measures can be broadly categorised into: (1) policy/guidelines, (2) online courses, (3) face-to-face workshops (contact time), (4) information dissemination via emails.

The NTU Policy on Research Integrity and Responsible Conduct of Research is a 15- minute online read that depicts the roles and responsibilities of researchers to uphold the highest standard of research integrity and ethical behaviour in the pursuit of research excellence and rigour. The online course is in the form of Epigeum RI course, which outlines how research should be designed, conducted and reported ethically. This 8-h course is concluded with a quiz of 50 questions which requires 80% of correct answer to pass. Researchers are expected to re-take the course every 3 years to keep up with updates and refresh their memory, which ties in with spaced repetition. Face-to-face workshops primarily refers to the Data Management Plan (DMP) workshop, which is a half day course to guide researchers on how to write a document delineating the data lifecycle from data collection to preservation. This workshop covers topics on the steps of data collection, processing, analysis, storage, access, sharing and archiving. Lastly, the NTU Research Integrity and Ethics Office produces and disseminates the Compass e-newsletter monthly to inform the research community about the current good research practices as well as ethical obligations with regards to human biomedical research and human tissue framework.

Despite some degree of overlap, the combination of these channels ensures good outreach and coverage of sufficient scope of RI education for different research sub-populations in the university. Aside from DMP workshops, the other channels of RI education are mostly self-guided, which have lower participation rates. Even though reading the NTU RI policy is mandatory, it is not possible to enforce it in totality. As for the Epigeum online course, researchers can still easily game the course despite the inclusion of quizzes in the program. These limitations can impede NTU’s progress in educating researchers on RI. Hence, there is a need to understand how current measures in NTU RI education can be improved to better engage their attention. This research aimed to assess the attractiveness of these RI education channels to the NTU research community and its subpopulations. The information gathered through this study will be useful for designing RI education programs that are more targeted and aligned with the preferences of researchers in NTU.

## Methodology

### Survey design

#### Pilot study and survey dissemination

An online survey was designed on Qualtrics platform and sent out to selected NTU research members as a pilot study to test the validity of the survey (see Additional file 1). Survey questions were designed to obtain information on their preference for RI education modes as well as their experience with each of the RI education channels offered by NTU. Free response questions were also included to acquire a more nuanced understanding of their perceptions. Based on the feedback from the pilot study, more information (e.g., screenshots and links) was added to help trigger respondents’ memory of the different modes of education. Another issue raised in the pilot study was survey fatigue, which led to the removal of four questions to improve accuracy and response rates. Structurally, the order of the questions was also rearranged to improve the flow of the survey. Majority of the questions went through minor edits (e.g., added/removed options, included text entry option, improved phrasing of question and Likert options). The finalised survey was disseminated to the NTU research community via emails with an embedded poster to explain the study in brief. Two reminder emails were sent, each a month apart to boost response rate. Invited participants included research students (Masters or PhD), research staff and faculty members from all colleges in NTU. Although the target group was originally limited to researchers from Lee Kong Chian School of Medicine (LKC), College of Science (CoS), and National Institute of Education (NIE), it was later expanded to include College of Humanities and Social Sciences (CoHASS), Nanyang Business School (NBS) and College of Engineering (CoE) to increase sample size. As the emails were sent out via coordinators in the respective colleges, it was not possible to ascertain how many researchers were reached out to.

Participation in the survey was voluntary and data were collected and analysed anonymously. No IP addresses were collected in the survey. This was emphasized in both the email and the survey form. Informed consent was also taken online before the respondents commenced the survey (NTU-IRB Ref No: IRB-2020-03-056). After the survey closed, the data were analysed using various statistical packages in R.

#### Survey sections

The survey had 3 sections. The first section collected data regarding respondents’ demographic profile (e.g., age, designation, school, years of research experience). In the next section, the respondents completed a ranking exercise on their preference for different channels of RI education. All NTU researchers have access to RI education in four different forms, comprising the Epigeum online course, NTU Research Integrity Policy, Data Management Practice (DMP) workshops and the COMPASS e-newsletters. COMPASS is a monthly online newsletter published by NTU Research Integrity and Ethics Office (RIEO) and is disseminated via emails to communicate matters related to good research practice. The respondents had to rank the 4 NTU RI education modes, but they may choose not to rank the modes which they were not familiar with.

**Fig. 1:**
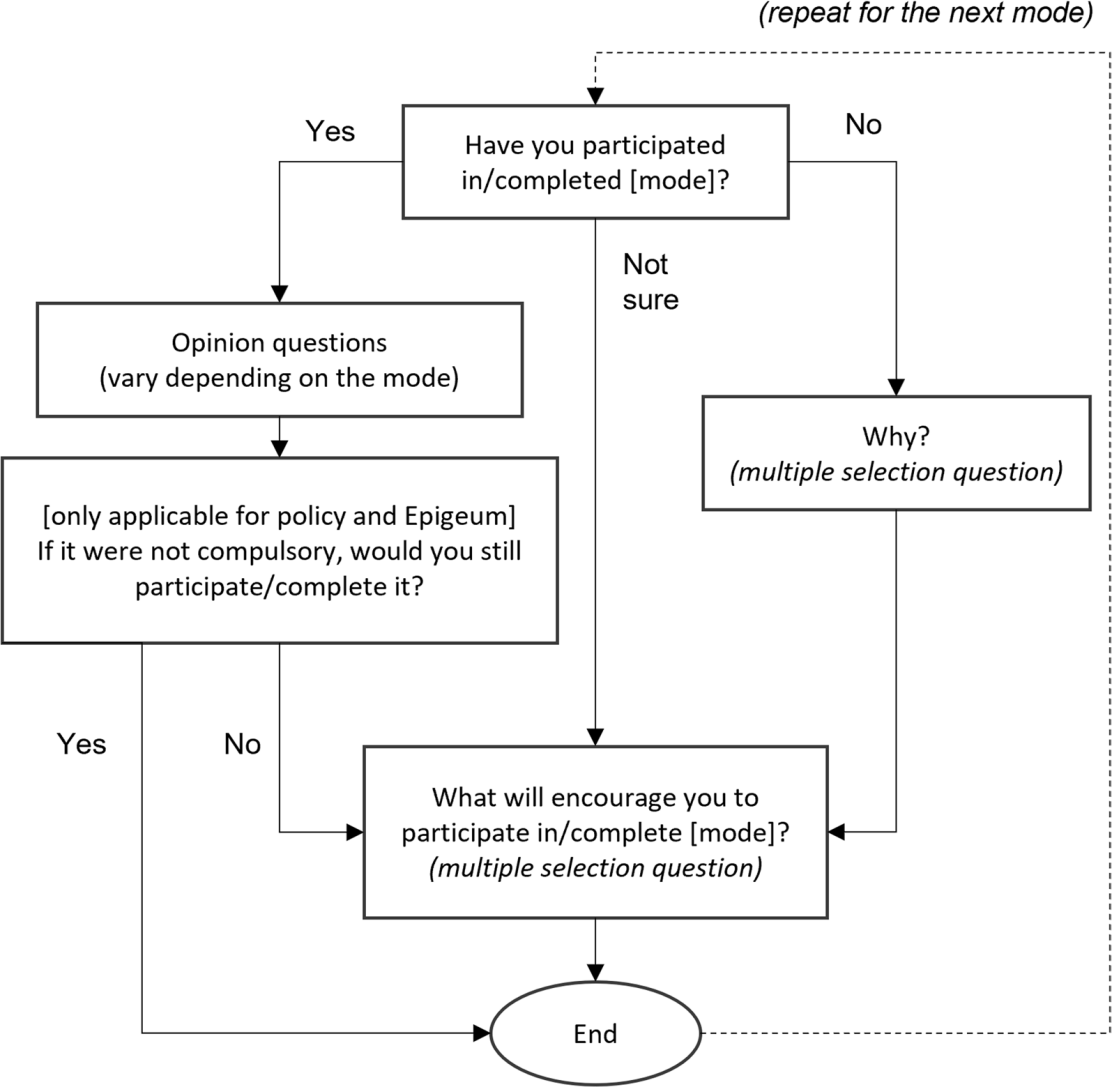
Flow chart of questions in third section of survey

In the final section, the respondents were surveyed about their experience in each mode of RI education that they haved participated in. Hence, those who had completed some RI education would have more questions to answer than those who had not done any RI education. Respondents who indicated that they had not participated in the mode previously were to select or state their reasons for not doing so in the survey. Figure 1 illustrates the flow of questions in this section.

### Statistical analyses

The sample population was divided into subpopulations for statistical analysis, based on the profiles that might differentiate preferences for RI education channels i.e., (1) Field (STEM/non-STEM), (2) Roles in the community (Faculty/staff/student),^1^ (3) Age (< 30 years old, 30–50 years old, > 50 years old), and (4) Native/non-native English speakers. Although the respondents were from six different colleges, their domain of research was coded as STEM/non-STEM. While preferences in the subfields were not fully homogeneous, the analysis of data according to STEM/non-STEM fields took into consideration broad differences in pedagogy and research methods between these two academic domains. LKC, CoS and CoE were classified as STEM fields while CoHASS, NBS and NIE were classified as non-STEM fields.

#### Ranking question

Since the ranking question only required respondents to rank the RI education modes that they were familiar with, there were missing values when respondents chose not to rank certain modes. Hence, additional steps were taken to prepare the ranking data for analysis. Firstly, responses where only a single RI education mode was ranked were excluded from analysis as they provided no information about the respondent’s preference. Next, modes which were not ranked were imputed with the mean rank of that mode. Using the mean values for imputation minimised the impact on the central tendency with each mode and preserved the ranking order. After imputation, the data set was complete with no missing values.

Overall preferences of the entire sample population across the RI education modes were compared using Friedman test. This test is the non-parametric equivalent to the repeated measures analysis of variance (ANOVA) test, which is suitable as every respondent had a ranking for every mode of instruction. The null hypothesis was that there were no differences (in ranking) between the modes. If the null hypothesis was rejected, the Nemenyi post-hoc test was conducted to identify pairwise differences.

Analysis of RI education preferences was also conducted within each of the four subpopulations i.e., STEM/non-STEM, Age groups, Designations, and Native/non-Native English speakers. Friedman test (and Nemenyi post-hoc test) was conducted for each subpopulation. Kruskal Wallis H (KW) or Mann Whitney U (MW) tests were used to compare preferences between subpopulations for each mode [18, 19]. MW test was conducted if there were 2 groups in the subpopulation. KW test ws conducted if there were more than 2 groups. If the null hypothesis was rejected, the KW test was followed up by Dunn’s test which reveals where the differences were.

#### Participation rates

The Chi-square test was used to compare participation rates across the four subpopulations for each of the four modes of RI education. Responses from those who joined NTU for less than a year and ‘not sure’ responses were omitted from the analysis of participation rates to maximise accuracy of results.^2^ Results from subsequent multiple selection questions were discussed, but no statistical tests were conducted due to the open-ended nature of the data.

#### Opinion questions

Most of the opinion questions fell into one of two categories of ‘Structure’ and ‘Impact’ (Table 1). Multiple-choice questions (MCQ) were transformed to dichotomous variables by combining similar choices. Likert scale items were converted to a numeric variable, ranging from 0 to 3. The first category, ‘Structure’, surveyed respondents on how the RI education modewas structured. Q1 and Q2 were analysed individually using Chi-square tests while Q3 and Q4 was analysed using KW test (and Dunn’s test). Questions in the ‘Impact’ category were self-reported assessments of how useful the RI education mode is to the individual and their community. They were summed together to form scores and these scores were compared across modes using KW test (and Dunn’s test).

**Table 1:** Derived parameters from the opinion question

No	Question type	Questions^a^	Mode of education^b^
*Structure*			
Q1	MCQ	How reasonable is the time needed to complete […]?	All
Q2	MCQ & open-ended^c^	Do you think the amount of information provided in […] should be changed?	All
Q3	Likert	How would you rate the flow of information provided on […]?	All
Q4	Likert	How would you rate the user-friendliness of […]?	Only policy and Epigeum
*Impact*			
Q9	Likert	How useful is the information provided?	All
Q10	Likert	How useful is […] in promoting good research practices in your school/workplace?	All
Q11	Likert	How interested are you in learning more about research integrity after completing […]?	All

^a^There are more questions than what is listed in this table. Only questions from third “Results” section that were analysed are included. Due to the scope of the paper, some questions were left out

^b^This column specifies the mode of RI education that was asked by question. Only questions that are analysed are included

^c^Respondents were allowed to elaborate on their chosen option

For all the questions, the ‘Not applicable’ responses were replaced with the mode of the responses (i.e., most frequent value)

#### Open-ended questions

At the end of each section, the respondents were also asked about how the specific RI education mode can be improved. There were also several questions where respondents may choose to elaborate their responses if suitable options were not presented in the choices of structured answer. For example, for the question “what will encourage you to participate in [mode]?,” a few choices were provided but respondents could also list down their own answers. Most of the open-ended responses were general feedback and possible improvements on the mode. Hence, open-ended responses for different questions were compiled together for simplicity. Due to limited data, a simple frequency table was constructed to describe their responses and no further analyses were conducted. However, the open-ended responses will be discussed alongside the other results in the discussion section.

## Results

### Participant profile

The study recruited 272 participants, but 30 participants were excluded from analysis due to incomplete data.^3^ The final set of data had 242 researchers from various disciplines in NTU, representing an 89% inclusion rate in the survey. Of the 242 NTU researchers, 209 of them finished the entire survey while the rest quitted halfway. Table 2 provides a breakdown of the demographics of respondents surveyed.

**Table 2:** Summary statistics of sample population

Profile	N = 242^a^
*Gender*	
Female	119 (49.2)%
Male	115 (47.5)%
Prefer not to say	8 (3.3)%
*Age*	
< 30 years old	100 (41.3)%
30–50 years old	121 (50.0)%
> 50 years old	21 (8.7)%
*Designations*	
Faculty member	43 (17.8)%
Research staff	78 (32.2)%
Research student	121 (50.0)%
*Native language*	
English	159 (65.7)%
Others	83 (34.3)%
*Field*	
non-STEM	94 (38.8)%
STEM	148 (61.2)%
*College*	
NBS	16 (6.6)%
CoHASS	29 (12.0)%
NIE	49 (20.2)%
CoE	45 (18.6)%
CoS	66 (27.3)%
LKC	37 (15.3)%
*Research experience (years)*^a^	5 (3, 10) years
*Research experience in NTU (years)*^a^	2.2 (1.0, 4.0) years

^a^n (%); Median (IQR)

Among the respondents, nineteen (7.9%) felt that learning or getting information on research integrity is not important to them. Ten of them were PhD students, of which nine were < 30 years old. Majority from this group are from STEM fields (CoS: 9, CoE: 5, CoHASS: 2, LKC, NBS, NIE: 1). In the follow-up multiple selection question, sixteen of these respondents selected the option ‘I have a high awareness of research integrity and do not need to know more,’ and four of them believed that ‘research integrity cannot be learnt or trained’.

Among respondents who reported that learning or getting information on research integrity were important to them (n = 223), 76.2% indicated that ‘research integrity is essential for scientific advancement’ and only 10.3% felt that they ‘had a low awareness of research integrity and would like to find out more’. These results showed that a large proportion of the research community recognise the importance of RI education as part of their professional practice in conducting research.

### Participation rates

**Fig. 2:**
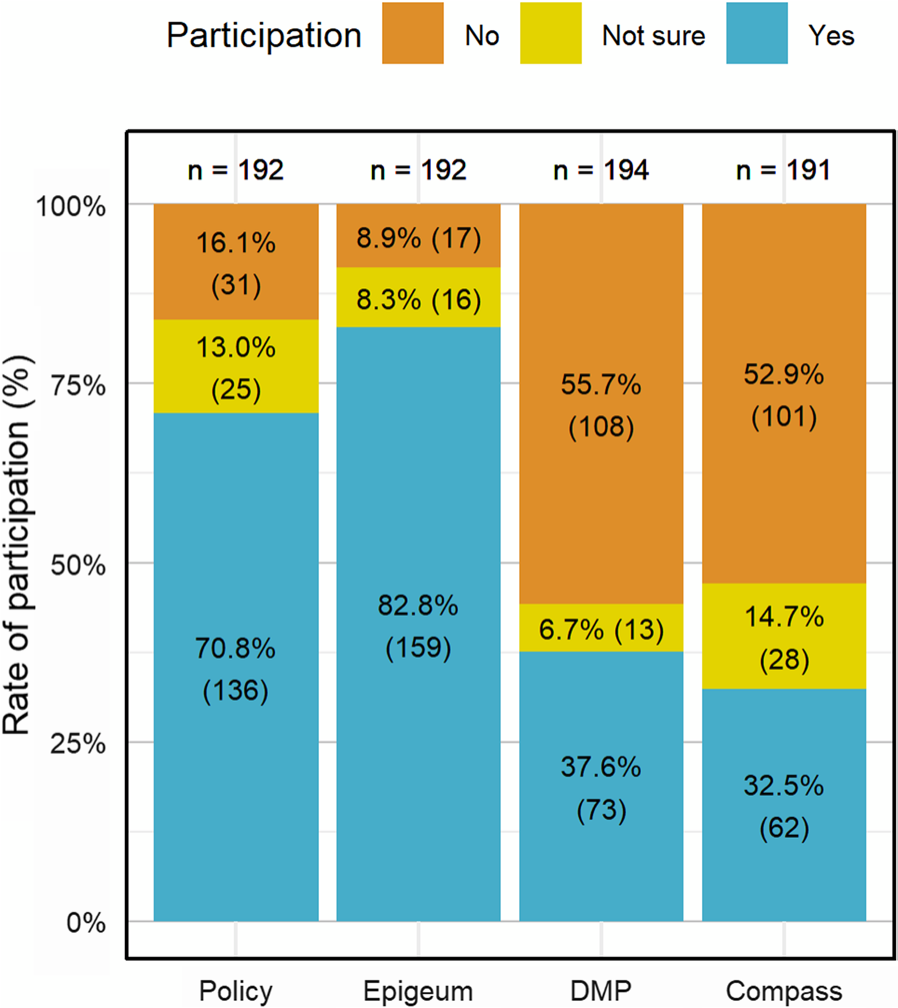
Participation rates across RI education modes. Respondents with less than a year of research experience in NTU were excluded. Sample size for each mode of education differs as the questions were randomized and not all respondents completed the survey

**Fig. 3:**
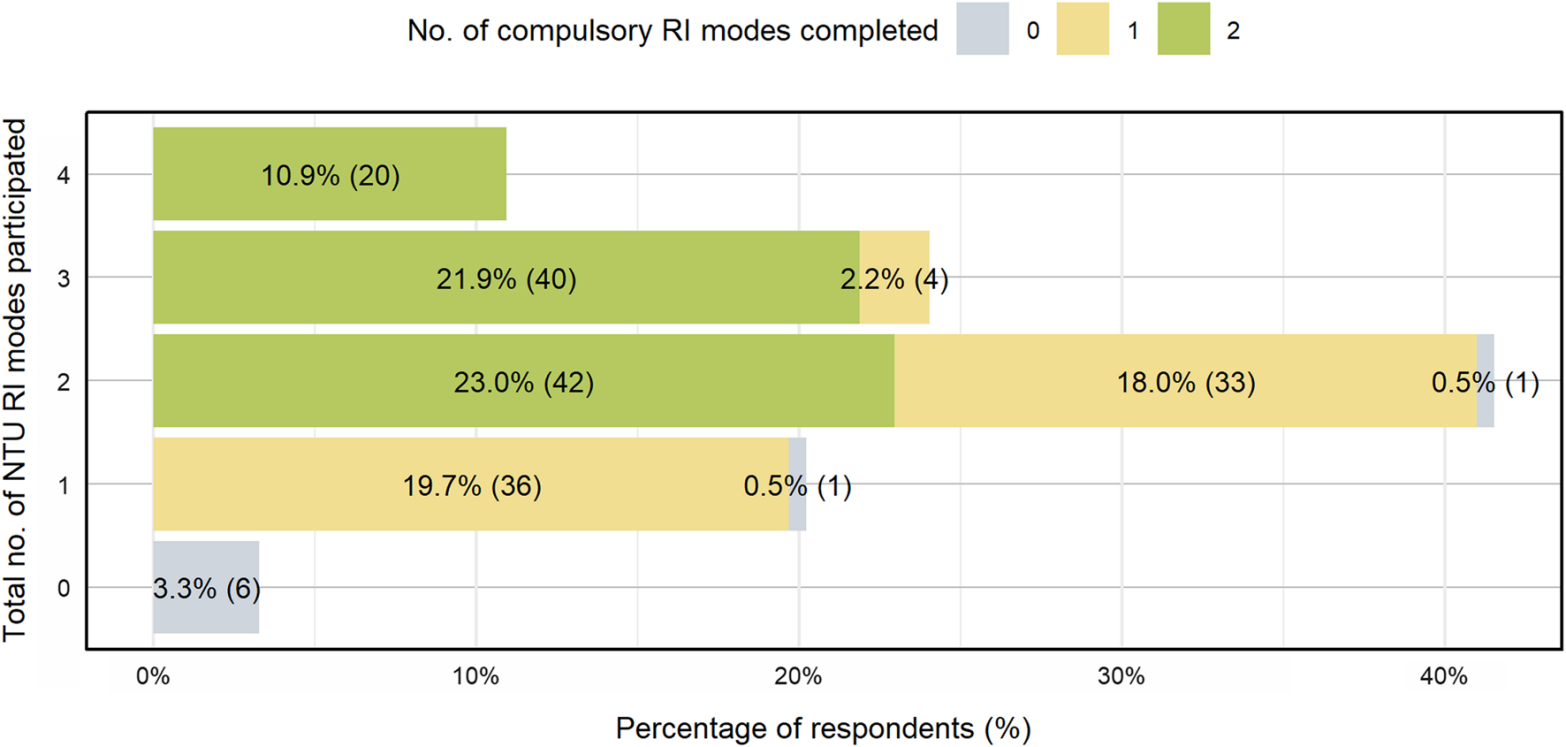
Total no. of NTU RI education modes participated before the survey (N = 183). Respondents with less than a year of research experience in NTU were excluded. Some respondents did not complete the survey and thus information on their participation in the RI education modes were incomplete. Their responses were filtered out as well

Figure 2 shows the participation rates of the various Research integrity modes of instructions. As the NTU Research Integrity policy and Epigeum course are compulsory, the participation rate for these channels were significantly higher. There was also a sizable proportion of “not sure” responses, especially for ‘Policy’ and ‘Compass’. Results from Fig. 3 shows that majority of the respondents (76.4%) have participated/completed at least two RI modes before the survey. These data indicate that regulating RI education participation is effective for ensuring high participation rates.

Results from Chi-square tests on participation rates of RI education modes across various subpopulations are presented in Table 3. Among the different designations, participation rates for ‘Compass’ decreased from faculty members (55%) to research staff (42%) and finally to research students (28%) (*p* < 0.05). Participation rates for ‘DMP’ also varied significantly across the different age groups (*p* < 0.05), showing an increasing trend with age. A plausible explanation is that faculty members are required to attend the DMP workshop for submission of proposals and faculty members tend to be older. However, ‘DMP’ participation rates did not differ significantly across different designations. The uneven sample sizes of the three different age groups may have further contributed to the positive chi-square result.

**Table 3:** Chi-square test results for participation rates of RI education modes (community/age/field/English)

	Policy	Epigeum	DMP	Compass
Community	0.262	0.0637	0.108	**0.0269**
Age	0.679	0.626	**0.0233**	0.192
Field	0.703	0.656	1.00	1.00
English	0.380	0.345	0.259	0.624

Values in boldface denote significant differences (*p* < 0.05)

### Opinion questions

#### Structure of each RI education mode

**Fig. 4:**
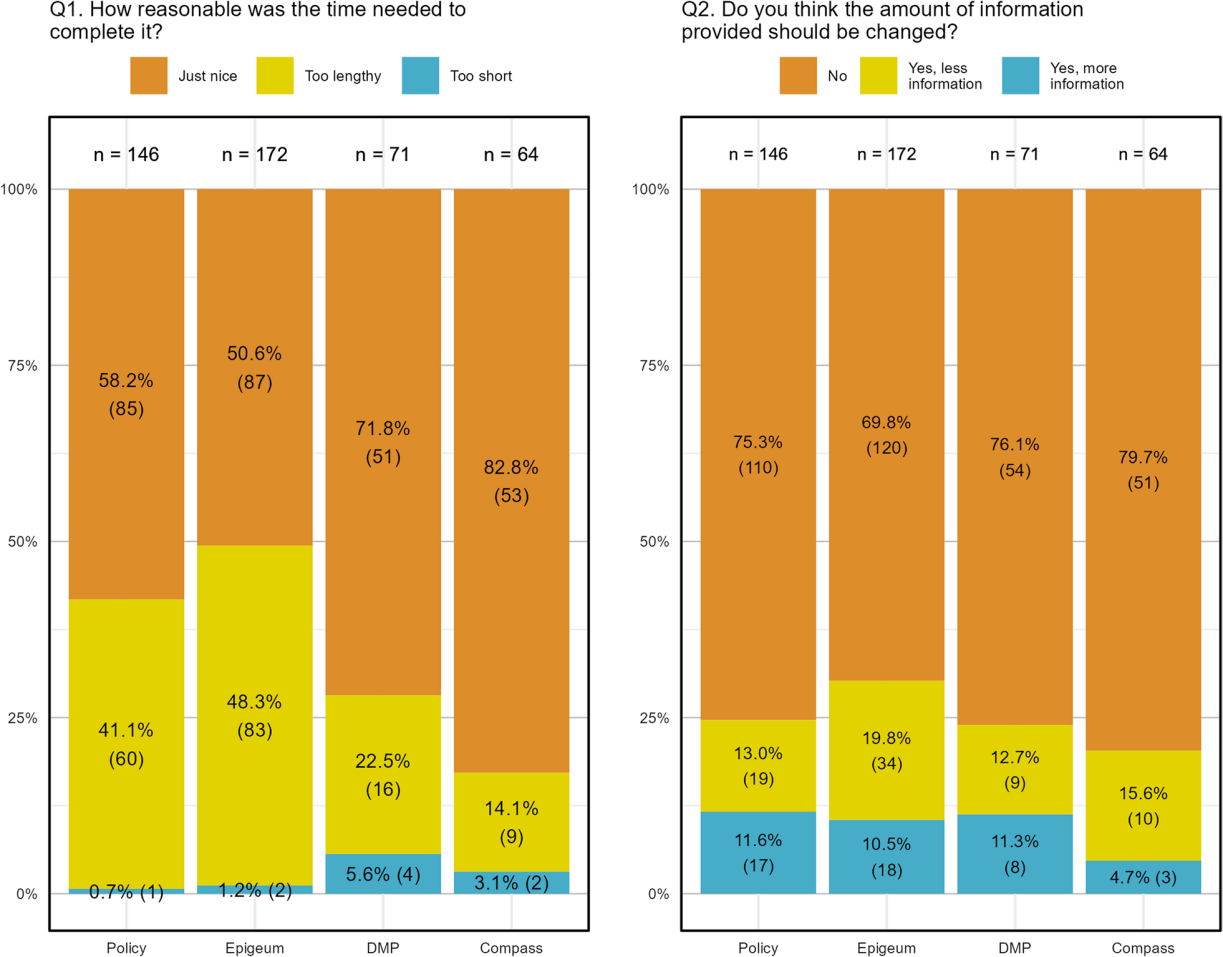
Responses regarding duration of and amount of information in RI education modes. These opinion questions were only presented to respondents who have completed the particular mode of education. Hence, sample size differs across different modes of education

Figure 4 depicts the responses to questions regarding duration and amount of information for each mode respectively. There were 70% and 80% of respondents who were satisfied with the amount of information provided. Preference for more and less information (Q2) was quite equally distributed for all RI education modes. The Chi-square tests showed no significant difference in the responses for Q2 across the different modes of RI education (*p* > 0.05). However, there was significant difference for Q1 responses (*p* < 0.05). It is evident from Fig. 4 that compared to other modes, a larger proportion of respondents (40%–50%) found ‘Policy’ and ‘Epigeum’ too lengthy, but this is an opinion question that should be weighed carefully with perceived educational effectiveness.

For a Likert item question regarding flow of information (Q3), results from the KW test suggests that there was no significant difference between the modes of education (*p* > 0.05). User experience (Q4) of ‘Policy’ and ‘Epigeum’ were also rated similarly (*p* > 0.05).

#### Impact of each RI education mode

Impact scores for all four modes of RI education (‘Policy,’ ‘Epigeum,’ ‘DMP,’ and ‘Compass’) were compared using KW test. There was no significant difference in impact scores between the different modes of education (*p* > 0.05). For the most part, impact scores also did not differ within the designations, fields, and age groups for each mode of education. However, there was a significant effect for ‘Epigeum’ showing that faculty members had a lower impact score than both staff and students.

#### Reason for not participating in RI education modes

**Fig. 5:**
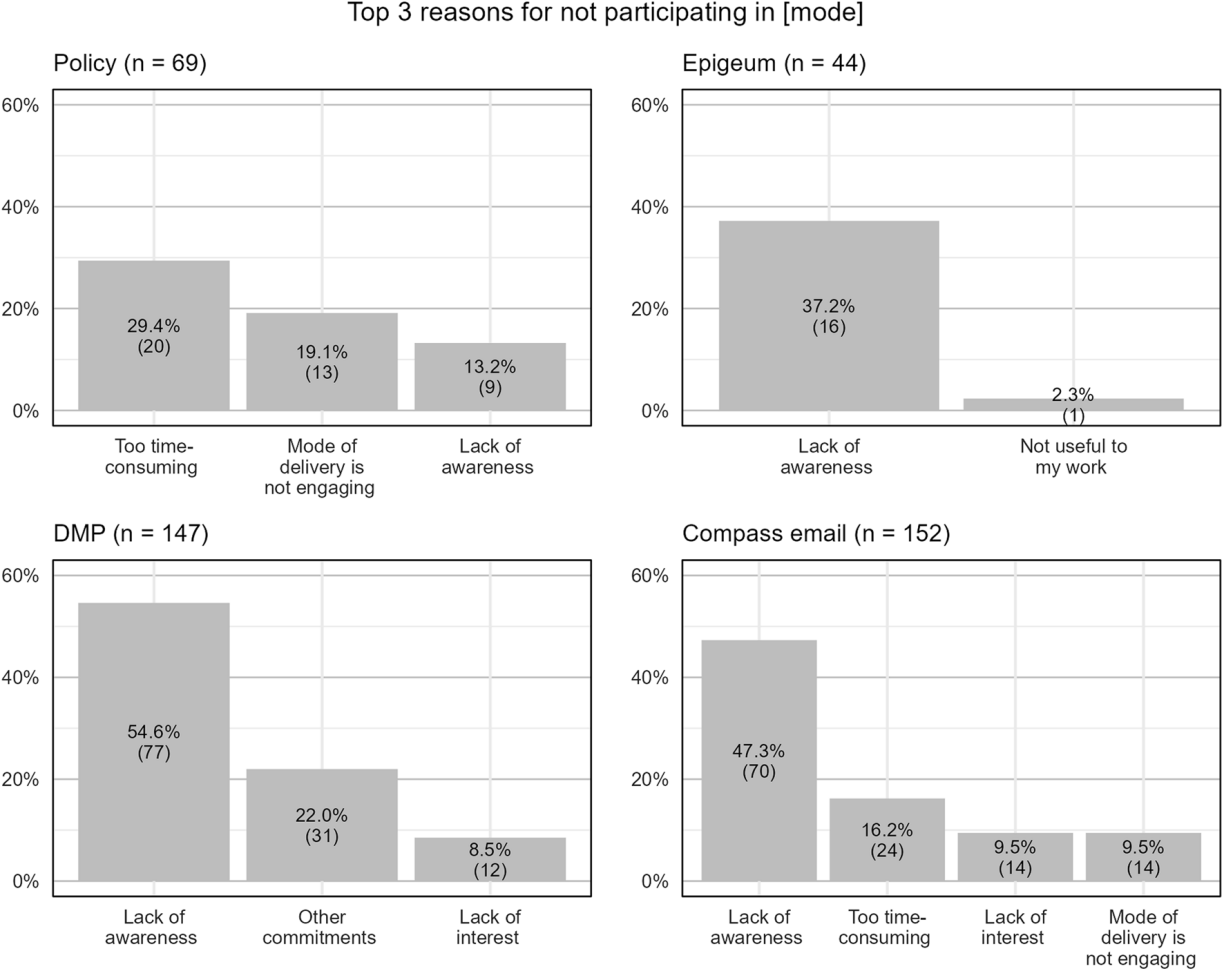
Top three reasons for not participating in RI education modes. This question is a multiple selection question (i.e., more than one option can be selected). Respondents may also opt to not select any of the options. Hence, numbers in the bars do not add up to the sample size cited for each graph

For respondents who did not participate in the RI education modes, there were follow-up questions to ascertain their reasons for not doing so. The “lack of awareness” was among the most cited reasons for not participating in all the modes of RI education and was the top reason for the modes of ‘Epigeum,’ ‘DMP’ workshop and ‘Compass’ e-newsletters (Fig. 5). For ‘Policy,’ the top reason for non-participation was that “it takes too much time to read,” which echoed the sentiments of those who read the RI policy (Fig. 4).

However, this was not the case for ‘Epigeum.’ Although many (48.3%) who completed the course felt that it was too lengthy, the duration of the programme was not among the top reasons for non-participation. It was also puzzling that NTU researchers were unaware about ‘Epigeum,’ as they are required to complete the online course and pass a quiz. Of the sixteen respondents who were unaware of ‘Epigeum,’ seven respondents had less than a year of experience in NTU.

**Fig. 6:**
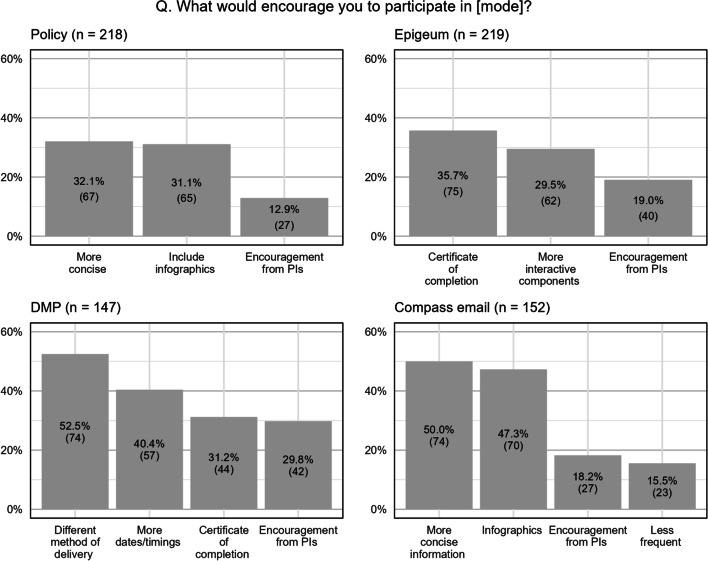
What would encourage you to participate in [RI education mode]? PI refers to Principal Investigator. This question is a multiple selection question (i.e., more than one option can be selected). Respondents may also opt to not select any of the options. Hence, numbers in the bars do not add up to the sample size cited for each graph

Results for a separate multiple selection question ‘What would encourage you to participate in [mode]?” are shown in Fig. 6. For ‘DMP,’ most respondents expressed interest for different delivery methods as well as more dates/timings. These responses reflected the desire for more flexibility and options.

Approximately half (47.3%) of the respondents prefer Compass e-newsletters to be more concise and include infographics. Receiving a certificate of completion was a popular option for ‘Epigeum,’ which also happens to be the mode that requires the most effort.

### Open-ended questions

Open-ended responses were compiled into a frequency table for each RI education mode (see Additional file 2). Generally, most respondents felt that the content could be more concise. For DMP workshop, several respondents believed that data management practices are not necessary and are merely a hindrance to their research. Some respondents also felt that information presented in the DMP workshop could be accessed through the webpage. Similarly, some respondents pointed out that Compass emails are just one of the many mass emails from the school and are not a priority. For ‘Policy’ and ‘Epigeum,’ both education modes could be improved by being more concise and enhancing the user interface.

#### NTU ranking

**Fig. 7:**
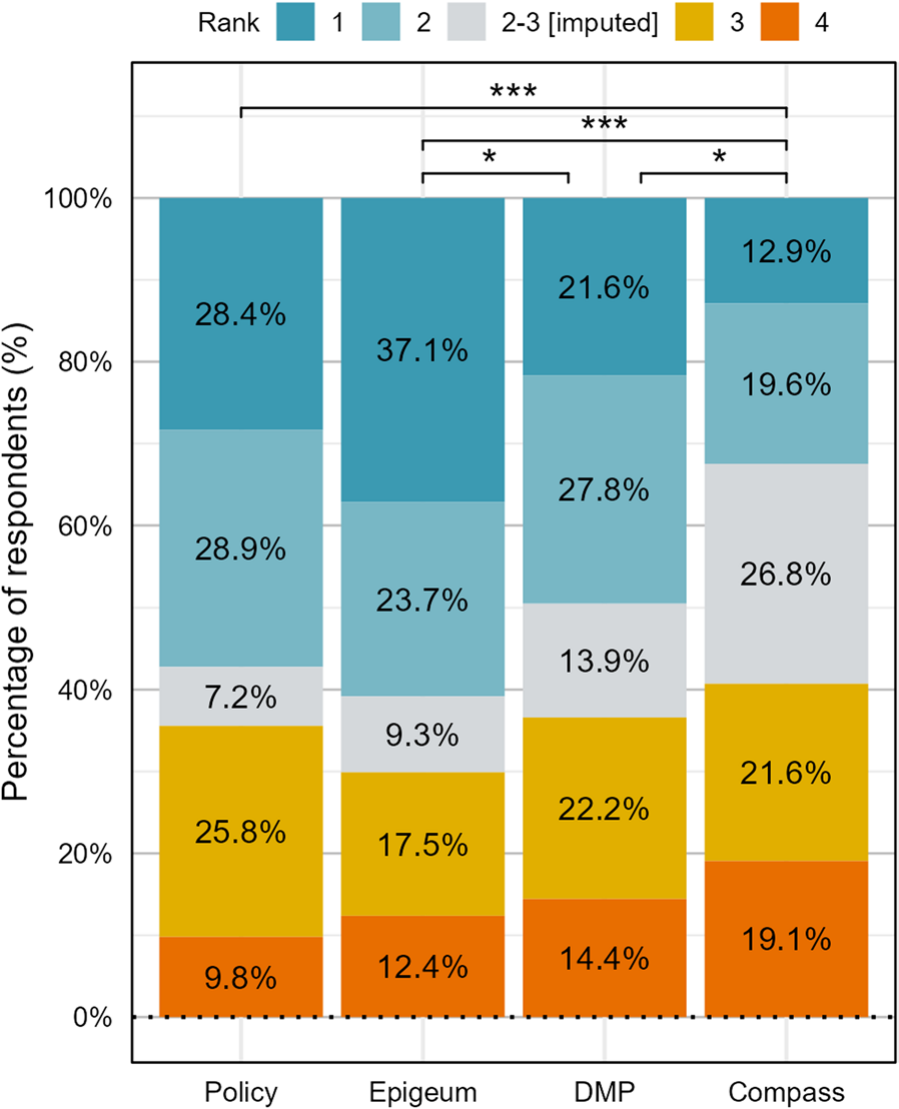
Ranking of preferences across NTU RI education modes (N = 194). *P* value for Friedman test was below 0.05, hence Nemenyi post hoc test was conducted. Results of the post hoc test are illustrated with significance bars (*p < 0.05, **p < 0.01, ***p < 0.001). ‘Compass e-newsletter’ was ranked the lowest compared to all other modes (*). ‘Epigeum’ was also ranked higher than ‘DMP workshop’ (***)

Though all 205 non-LKC respondents completed the ranking question, eleven respondents only ranked a single RI education mode. Hence, those responses were filtered out and 194 respondents were analysed (205—11 respondents). There was a significant difference in the rankings for each NTU RI mode (*p* < 0.05). Hence the post-hoc test was conducted. ‘Compass’ was the least preferred RI mode (*p* < 0.05) and ‘Epigeum’ was favoured over ‘DMP’ (*p* < 0.05) (Fig. 7). ‘Epigeum’ appeared to be the most preferred mode with 37.1% of respondents ranking it their first choice. There were 26.8% of respondents who did not rank ‘Compass,’ compared to between 7.2% and 13.9% for the other modes. This shows that ‘Compass’ is not well received by many in the research community.

There was no significant difference in preference for each of the RI education modes across the different subpopulations (*p* > 0.05) which suggests that preferences are relatively homogenous within the subpopulations.

Friedman tests for each subpopulation were conducted and most analyses yielded significant differences (*p* < 0.05). Results for the Nemenyi post hoc test are shown in Table 4. Two subpopulations (‘Faculty Members’ and ‘> 50 years old’) had similar ranking across the four RI education modes (*p* > 0.05), making the post hoc test unnecessary. These two subpopulations also had the smallest sample size (Faculty = 39; > 50 years old = 19) and thus, lower statistical power.

**Table 4:** Nemenyi post hoc test results for NTU ranking (community/age/field/native language)

	Policy	DMP	Compass	Policy	DMP	Compass
**Subpopulations**	**Research staff (n = 48)**			**Research student (n = 107)**		
DMP	0.436	NA	NA	0.989	NA	NA
Compass	**0.0362**	0.636	NA	**6.79E−03**	**0.0188**	NA
Epigeum	0.893	0.119	**3.77E−03**	0.548	0.355	**3.12E−05**
**Subpopulations**	Below 30 years old (n = 84)			30–50 years old (n = 91)		
DMP	0.975	NA	NA	0.256	NA	NA
Compass	0.0584	0.156	NA	**8.69E−04**	0.206	NA
Epigeum	0.306	0.137	**1.30E−04**	0.968	0.513	**4.81E−03**
**Subpopulations**	**STEM (n = 102)**			**Non-STEM (n = 92)**		
DMP	0.793	NA	NA	0.699	NA	NA
Compass	0.103	0.527	NA	**1.18E−03**	**0.0428**	NA
Epigeum	0.459	0.0797	**1.05E−03**	0.996	0.554	**4.73E−04**
**Subpopulations**	**Native English speakers (n = 126)**			**Non-native English speakers (n = 68)**		
DMP	0.0995	NA	NA	0.885	NA	NA
Compass	**6.64E−05**	0.154	NA	0.671	0.246	NA
Epigeum	0.995	0.0544	2.11E−05	0.310	0.752	**2.23E−02**

Values in boldface denote significant differences (*p* < 0.05)

‘Epigeum’ was preferred over ‘Compass’ for all the post hoc tests (*p* < 0.05). 'Policy’ was preferred over ‘Compass’ for the research students, 30–50 years old, non-STEM, and native English spearkers subpopulations. For non-STEM and research student subpopulations, ‘DMP’ was also preferred over ‘Compass,’ thus making ‘Compass’ the least preferred RI education mode. Although analysis of the entire sample population revealed that ‘Epigeum’ was ranked higher than ‘DMP,’ this preference did not appear in any of the subpopulation analysis.

## Discussion

About 92% of NTU research community subscribed to the importance of RI education as part of their roles in the university. These results not only reflect a high degree of buy-in to RI as a good practice, but also reflected the positive attitude of researchers in NTU towards RI education. More than 75% felt that it is part of their professional roles to participate in RI education and about 14% felt the need for more education to increase their knowledge in good RI practices. However, research integrity is a broad topic. Though most researchers adopt a positive stance towards RI education, they may not understand the point of a full-blown audit or having data management plans. These sentiments are apparent in the qualitative feedback collected. Nevertheless, the high subscription rate to RI education provides NTU with a strong foundation to promote participation in RI education, especially in education modes that are not compulsory (‘Compass,’ e-newsletter and ‘DMP’). The opportunities and potential strategies to improve participation rates are discussed below.

For the analysis of the overall sample population, ‘Compass e-newsletter’ was ranked the lowest. Though participation rates for Compass e-newsletters and DMP workshops are comparable, more respondents chose not to rank Compass e-newsletters. This suggests that despite reading the e-newsletters, many felt that they were unfamiliar with it. A likely explanation is that the e-newsletter was not memorable enough and respondents skimmed through the newsletter without retaining anything. Compass e-newsletters are often not a priority and topics in each issue of Compass e-newsletters/email may be too generic to be attractive to individual researchers. The issue of competing priorities is not unique to Compass e-newsletters. Resistance to RI education due to competing priorities was also highlighted at Labib and colleagues [20]. Qualitative feedback for Compass e-newsletters was mixed, with most thinking that it is a spam email that clutters their inbox, while a few respondents appreciated it for the timely updates. The consensus is that the e-newsletter is too wordy and unengaging, and that it can be improved with more concise information and infographics in presentation.

‘Epigeum’ was favoured over ‘DMP,’ however, this was not reflected in the subpopulation analysis. Yet, there was a common thread in the qualitative feedback for ‘DMP,’ where several non-STEM researchers felt that the DMP workshop catered mostly to STEM researchers and was of little relevance for non-STEM researchers. This sentiment was only present among non-STEM researchers. On the other hand, some STEM researchers responded that DMP workshops should allocate more time for in-depth discussion about specific questions. Unlike DMP workshops, the Epigeum course has various tracks that are tailored to different specialisations, which was better appreciated by the respondents. Hence, it would be beneficial if DMP workshops were similarly tailored to the different fields of research. As one respondent pointed out, more specialised information may be required as research activities get more specific. This is also in-line with existing research that RI education should be tailored to researchers’ discipline and level of experience [20, 21]. If possible, steps should be taken to further accommodate different learning styles.

The lack of awareness was the most cited reason for not participating in ‘Epigeum,’ ‘DMP,’ and ‘Compass.’ These results could explain the lower participation rates for Compass e-newsletters and DMP workshops as they are not compulsory and could be overlooked. Although the NTU Research Integrity Policy is compulsory reading for all researchers, there is no reliable way to ensure everyone reads it. However, Epigeum online course is a compulsory module that has a quiz after each section to ensure researchers pass all the necessary checkboxes. Those who were unaware of the course could either be left out by the system (i.e., technical issues) or forgot that they have already completed it. For the latter, the problem may lie in the Epigeum course itself because it is easy to skim through the online course and the quiz, which can be repeated until a satisfactory score is achieved. Hence, it is entirely possible for one to complete the Epigeum course without retaining much knowledge. As brought up in Sefcik et al.’s research, there is always the concern of them gaming the online courses [2].

Although most researchers acknowledge the importance of RI education, the compulsory RI education modes (‘Epigeum’ and ‘Policy’) enjoyed significantly higher participation rates of 70%–80%, compared with 32%–37% for the non-compulsory RI education modes (‘DMP’ and ‘Compass e-newsletter’). These findings suggest that regulatory mechanisms are still necessary to ensure higher participation rate in RI education, and thus core RI education content should be made compulsory in research/academic institutions. This is consistent with findings from Labib and colleagues that many researchers are still resistant to RI education. While mandating certain modes of RI education as a solution was agreed upon, there is concern that it will create even more resistance and become an obstacle to RI education instead [20]. Other strategies can be considered to boost participation rates in non-compulsory education modes, which has a baseline participation rate of < 38%. These would include improving the modes of delivery, and attractiveness of content and presentation. Based on the responses gathered, some possible ways of increasing participation in RI education include awarding a certificate upon completion of Epigeum and DMP, adding more interactive components in Epigeum course, having more flexibility in mode of delivery and dates of the DMP workshops, and having more concise information for Compass e-newsletters and RI policy.

‘Policy’ and ‘Epigeum’ were found to be too lengthy in presentation. Most respondents preferred these modes of delivery to be more concise in content and to utilise more infographics. At the same time, some suggested the inclusion of specific contents (e.g., conflict of interest, open data/open science topics) and more real-life examples in the delivery of the materials. Most researchers use the policy webpage as a guide or reference when needed. Few read the entire policy as it is lengthy and verbose. While the links were found to be useful for navigating to specific sections in the policy page, the entire webpage was unengaging. The branching of content hides information and lengthens the time needed to read the policy. There were suggestions to include a “search bar” and to have a more comprehensive FAQ section. Many of the suggestions to improve the browsing and searching experience further reinforced how researchers view the policy webpage as a reference instead of required reading. Of the existing RI education modes, ‘Policy’ is the simplest to access and is likely the first place that researchers consult when they require guidance. In turn, the policy webpage should point them to the right direction, through links or avenues of contact. Incorporating simple infographics and providing more user-friendly elements to the website will also make the policy a more useful reference tool for researchers.

Similarly, for the Epigeum online course, poor user interface and other technical issues were raised several times in the qualitative feedback. These factors negatively affected the user experience and made the course unnecessarily tedious. While the estimated time for completion was 8 h, the course has too many components and levels of information, which makes it difficult to accurately estimate the time required for each section. Furthermore, the courseware has many technical issues (e.g., videos not loading, broken links), which dampened the user experience. Others have found that students appreciated the use of more engaging techniques such as storytelling, entertaining scenarios, gamified quizzes to teach academic integrity related topics [22]. A similar approach could be taken to increase the appeal of the Epigeum online course.

### Limitations

Discussion of such a sensitive topic as research integrity might lead participants to respond more favourably than their actual state of belief. For example, for the question “Is learning or getting information on research integrity important to you?”, there is a possibility of self-reporting bias, consciously or subconsciously.

The survey was carried out during the COVID-19 pandemic. At that point, the DMP workshops had shifted to an online format and were conducted via zoom meetings. Hence, a selected group of respondents who attended the workshop during the pandemic experienced the workshop in a different format. In addition, the DMP workshop only educated participants on data management practices and is unlike the Epigeum online course. Hence, it will be erroneous to regard comparisons between these two education modes as face-to-face training vs online training in totality.

The survey was also conducted on NTU researchers and is specific to RI education in NTU. While results from this survey may be taken into consideration for designing RI education in other institutions, it should not be generalised. It is also important to note that this sample is neither exhaustive nor fully representative of the NTU research population.

## Conclusion

Each RI education mode takes a different approach in ensuring that NTU’s diverse research community upholds research integrity. NTU has established a strong foundation for RI education, with > 92% of researchers embracing the programmes offered by the university. Despite this high subscription rate, the data also highlighted areas of divergence and convergence in terms of preferences and responses to each of the education modes. For example, although ‘Epigeum’ and ‘policy’ enjoyed the highest participation rates, due mainly to institutional requirements, they can be more impactful by improving user experience e.g., user-friendliness, relevance (to specialisation), having concise information and better presentation of materials. The baseline of < 38% participation rate in non-mandatory RI education modes also provides the opportunity for targeted strategies to improve participation in these modes of RI education. Some of the suggestions from the respondents to improve self-driven participation included the use of infographics, having more concise information, and the issuing of certificates for course completion.

Taken as a whole, this study has allowed NTU RI agency to better understand the preferences of the research community in terms of RI education modes and delivery. These findings can also be a useful benchmarking tool for assessing the effectiveness of strategies to improve participation rates in the respective RI education modes. More importantly, this study provided a means for the research community to inform NTU on how RI education can be made more appealing to them. This set of data is an important reference for reviewing and designing RI education strategies to be more targeted and effective in promoting a positive culture for RI across the university. For other institutions with similar modes of RI education as NTU, the results from this study may be helpful for improving participation rates and presentation of RI education modes, to be more appealing, such as the use of infographics and more concise information.

## Supplementary Information

**Additional file 1.** Survey Instrument for the study.

**Additional file 2.** Summary of responses to open-ended questions in the survey.

## Data Availability

The datasets generated and/or analysed in the current study are not publicly available due to confidentiality of information. However, they can be made available from the corresponding author on reasonable request.

## Abbreviations

RI: Research Integrity
NTU: Nanyang Technological University
NIH: National Institute of Health
NSF: National Science Foundation
IRB: Institutional Review Board
CITI: Collaborative Institutional Training Initiative
LKC: Lee Kong Chian School of Medicine
CoS: College of Science
CoE: College of Engineering
CoHASS: College of Humanities, Arts, and Social Sciences
NBS: Nanyang Business School
NIE: Nanyang Institute of Education
GRPO: Good Research Practice Office
RIEO: Research Integrity and Ethics Office
KW: Kruskal Wallis H test
MW: Mann Whitney U test

## Footnotes

1 Masters and PhD students were coded as research students. Research assistants, research associates, research fellows and lab managers were coded as research staff. Lecturers and professors were coded as faculty members.

2 Note that these respondents are only excluded from the analysis of participation rates. Their responses for other questions are still included and analysed as despite them only joining recently, they might have completed various RI education modes already or may have insights that are worth analysing.

3 Participants who did not complete “Methodology” section of the survey (i.e., ranking questions) were excluded.
