# Supplementary material for: Perception and reaction of Nanyang Technological University (NTU) researchers to different forms of research integrity education modality
Source: BMC Med Ethics. 2022 Aug 24;23:85. doi: 10.1186/s12910-022-00824-6 (PMC9400004; doi:10.1186/s12910-022-00824-6)
Supplement: Supplementary file 1 — Additional file 1. Survey Instrument for the study. [file 12910_2022_824_MOESM1_ESM.docx]

**Survey Questionnaires**

**Title:  Perceptions and reactions of NTU researchers to different forms of research integrity training methods** (NTU-IRB ref no: IRB-2020-03-056)    

You are invited to participate in a research study to understand preferences for research integrity learning or communication modes. Your response will help us to improve research integrity education and communication in NTU.  This study is conducted by Associate Professor Fabian Lim Chin Leong from Lee Kong Chian School of Medicine, Nanyang Technological University Singapore    

This study will take approximately 10-15 mins of your time and is anonymous. You will be asked to complete an online survey.  Some profiling data e.g. job position will be collected. Once the survey is submitted, the data cannot be deleted as it is not identifiable. All data collected will be kept in a secure network drive and will be uploaded onto an open access data repository upon project completion.  A total number of 300 participants from NTU research community will be recruited for this research. 

Your participation in this study is completely voluntary, and you have the right to withdraw from the study at any time without any penalty. If you do not wish to complete this survey, just close your browser.  Your participation in this research will be kept confidential, and data will be averaged and reported in aggregate. Your responses will be anonymous and IP addresses will not be collected to guarantee complete anonymity. Possible outlets of dissemination may be through a publication in a scientific journal.

Although your participation in this research may not benefit you personally, it will enable us to refine and improve each method of research integrity training to appeal to the research community. There will be no compensation provided. There are no risks to individuals participating in this survey beyond those that exist in daily life.    
 
If you have questions about this project, you may contact:
a. PI: Associate Professor Fabian Lim Chin Leong, fabianlim@ntu.edu.sg, 6592 3931 
b. Co-PI: Associate Professor Yusuf Ali, yusuf.ali@ntu.edu.sg, 6592 3930 
c. Collaborator: Celine Lee Ser Lin, celine.lee@ntu.edu.sg, 6592 3982
d. Research Assistant: Chua Yi Lin Jolene, jolene.chuayl@ntu.edu.sg, 6904 7236  

This project has been reviewed and approved by NTU-Institutional Review Board.
Questions concerning your rights as a participant in this research may be directed to the NTU-IRB at IRB@ntu.edu.sg or call 6592 2495.    
 
Please print a copy of this consent form for your records, if you so desire.     
   Please indicate your choice of participation below:

- I have read and understood the above consent form. I certify that I am 21 years old or older and, by clicking the button to enter the survey, I indicate my willingness to voluntarily take part in the study.
- I do not wish to participate in this study.

**Do not proceed with the survey if you do not wish to participate in this study**

**Survey starts here**

Q1 Which college are you from?

- College of Science
- Lee Kong Chian School of Medicine
- National Institute of Education
- Nanyang Business School
- College of Humanities, Arts and Social Sciences
- College of Engineering
- S. Rajaratnam School of International Studies

Q2 What is your sex?

- Male
- Female
- Prefer not to say

Q3 Which age group do you belong to?

- Below 30 years old
- 30 - 50 years old
- Above 50 years old

Q4 Is English your primary language?

- Yes
- No

Q5 What is your current role in research?

- Masters student by coursework
- Masters student by research
- PhD student
- Research assistant
- Research associate
- Research fellow
- Lab manager
- Lecturer
- Senior Lecturer
- Assistant professor
- Associate professor
- Professor
- Others: ________________________________________________

Q6 How many years have you been studying/working in a research-related role?

________________________________________________________________

|  |  |
| --- | --- |

Q7 How many years have you been studying/working in NTU in a research-related role?

________________________________________________________________

|  |  |
| --- | --- |

Q8 Please rate your preference on the following general modes of communication for learning or getting information on research integrity. *(1 = most preferred, 5 = least preferred)*

|  |
| --- |
| ______ Laboratory audits on research integrity and data management practices |
| ______ Policies and guidelines available online |
| ______ Face-to-face workshops |
| ______ Information disseminated through emails |
| ______ Online courses |

|  |  |
| --- | --- |

Q9 Please rate your preference on the following modes of communication for learning or getting information on research integrity in NTU. If you are not familiar with a mode, please place it in the 'Not applicable' box. *(1 = most preferred, 5 = least preferred)*

| **Most preferred** | **Not applicable** |
| --- | --- |
|  |  |
| ______ NTU Research Integrity Policy | ______ NTU Research Integrity Policy |
| ______ NTU Library Data Management Plan workshops | ______ NTU Library Data Management Plan workshops |
| ______ THE COMPASS newsletter by NTU Research Integrity and Ethics Office | ______ THE COMPASS newsletter by NTU Research Integrity and Ethics Office |
| ______ Epigeum Research Integrity online course | ______ Epigeum Research Integrity online course |
|  |  |

Q10 Is learning or getting information on research integrity important to you?

- No (Skip Q11)
- Yes (Skip Q12)

Q11 Why is learning or getting information on research integrity important to you? *(You may select more than one option.)*

- I have a low awareness of research integrity and would like to find out more
- Research integrity can be learnt or trained
- Research integrity is essential for scientific advancement
- Research integrity practices may become outdated over time
- Others: ________________________________________________

Q12 Why is learning or getting information on research integrity not important to you? *(You may select more than one option.)*

- I have a high awareness of research integrity and do not need to know more
- Research integrity cannot be learnt or trained
- Research integrity is not essential for scientific advancement
- Research integrity practices do not evolve over time
- Others: ________________________________________________

The questions in the following section is about NTU Research Integrity Policy. Information about these policies can be found in https://research.ntu.edu.sg/rieo/RI/Pages/NTU-Research-Integrity-Policy.aspx

Q13 Have you read the NTU Research Integrity Policy before?

- Yes (Skip to Q17 and Q18)
- No
- Not sure

Q14 Why? (You may select more than one option.)

- I am not aware of the policy
- It is not useful to my work
- It is not useful in promoting research integrity
- The policy takes too much time to read
- I am not interested in the topic
- The mode of delivery is not engaging
- Others: ________________________________________________

Q15 What would encourage you to read the research integrity policy webpage? *(You may select more than one option.)*

- More concise
- Include infographics
- Encouragement from PIs
- Others: ________________________________________________

Q16 NTU requires all members of the research community to read the NTU Research Integrity Policy when joining. If it is not compulsory, would you read the policy?

- Yes
- No

Q17 How reasonable is the time needed to finish reading the research integrity policy webpage?

- Just nice
- Too lengthy
- Too short

Q18 Currently, NTU requires the research integrity policy to be read once upon joining NTU.
How often do you think members of the research community should read the policy webpage?

- Only once upon joining
- Once every 3 years
- Once every 2 years
- Once a year
- As and when needed

Q19 Do you think the amount of information provided in the research integrity policy webpage should be changed? *(You may specify what information should be included/left out.)*

- No changes needed
- Yes, more information should be provided ________________________________________________
- Yes, less information should be provided ________________________________________________

Q20 How would you rate the flow of information provided on the research integrity policy webpage?

- Excellent
- Good
- Below average
- Poor

Q21 How would you rate the user-friendliness of the research integrity policy webpage?

- Excellent
- Good
- Below average
- Poor

Q22 How useful is the information provided in the research integrity policy webpage to you?

- Very useful
- Moderately useful
- Slightly useful
- Not useful

Q23 How useful is the research integrity policy webpage in promoting good research practices in your school/workplace?

- Very useful
- Moderately useful
- Slightly useful
- Not useful

Q24 How interested are you in learning more about research integrity after reading the research integrity policy webpage?

- Very interested
- Moderately interested
- Slightly interested
- Not interested

Q25 What would encourage you to read the research integrity policy webpage? *(You may select more than one option.)*

- More concise
- Include infographics
- Encouragement from PIs
- Others: ________________________________________________

Q26 How can the research integrity policy webpage be improved? *(Optional)*

________________________________________________________________

________________________________________________________________

________________________________________________________________

________________________________________________________________

________________________________________________________________

Q27 Have you read the following policies? *(Choose all that apply)*

- NTU Research Data Policy
- NTU Whistle Blowing Policy
- NTU Open Access Policy
- ⊗I have not read any of the above policies

The questions in the following section is about Epigeum Research Integrity online course. Information about the course is available in NTU Workday, [eLearning]: RI-ERIC

Q28 Which of the following online research integrity courses have you taken? Please choose the most recent course if you have taken more than one.

- 1. Epigeum Research Integrity Course (ERIC)
- 2. Epigeum Research Integrity Course - Student (ERIC - Student)
- 3. ERI701
- 4. Epigeum Research Integrity Course - Concise (ERIC - CONCISE)
- I have taken one before but cannot recall the title.
- I am not sure if I have taken one before.
- I have not taken one before.

*Skip Q29 and Q30 if your answer is* ***NOT*** *“I have not taken one before”*

Q29 Why? *(You may select more than one option.)*

- I am not aware of the course
- It is not useful to my work
- It is not useful in promoting research integrity
- The course takes too much time
- I am not interested in the topic
- The mode of delivery is not engaging
- Others: ________________________________________________

Q30 What will encourage you to complete Epigeum Research Integrity Online Course? (*You may select more than one option.)*

- Certificate of completion
- More interactive components
- Encouragement from PIs
- Others: ________________________________________________

Answer Q31 **ONLY** of your answer in Q28 is “Epigeum Research Integrity Course - Concise (ERIC - CONCISE)”

Q31 Which track did you complete?

- Biomedical Sciences
- Natural and Physical Sciences
- Engineering and Technology
- Social and Behavioural Sciences
- Arts and Humanities

Q32 If Epigeum Research Integrity online course was not compulsory, would you complete it?

- Yes
- No

Q33 How reasonable was the time needed to complete Epigeum Research Integrity online course?

- Just nice
- Too lengthy
- Too short

Q34 Currently, certification for Epigeum Research Integrity online course is valid for 3 years.
How often do you think members of the research community should take the course?

- Once every 5 years
- Once every 3 years
- Once every year
- Only once upon joining
- As and when needed

Q35 Do you think the amount of information provided in Epigeum Research Integrity online course should be changed? *(You may specify what information should be included/left out.)*

- No changes needed
- Yes, more information should be provided ________________________________________________
- Yes, less information should be provided ________________________________________________

Q36 How would you rate the flow of Epigeum Research Integrity online course?

- Excellent
- Good
- Below average
- Poor

Q37 How would you rate the user-friendliness of Epigeum Research Integrity online course?

- Excellent
- Good
- Below average
- Poor

Q38 How would you rate the explanations for quiz answers? Please select "not applicable" if you did not read the explanations.

- Excellent
- Good
- Below average
- Poor
- Not applicable

Q39 How useful is the information provided in Epigeum Research Integrity online course to you?

- Very useful
- Moderately useful
- Slightly useful
- Not useful

Q40 How useful is Epigeum Research Integrity Online Course in promoting good research practices in your school/workplace?

- Very useful
- Moderately useful
- Slightly useful
- Not useful

Q41 How interested are you in learning more about research integrity after completing Epigeum Research Integrity Online Course?

- Very interested
- Moderately interested
- Slightly interested
- Not interested

Answer Q42 **ONLY** if answer in Q32 is “No”

Q42 What will encourage you to complete Epigeum Research Integrity Online Course? (*You may select more than one option.)*

- Certificate of completion
- More interactive components
- Encouragement from PIs
- Others: ________________________________________________

Q43 How can Epigeum Research Integrity Online Course course be improved? *(Optional)*

________________________________________________________________

________________________________________________________________

________________________________________________________________

________________________________________________________________

________________________________________________________________

The questions in the following section is about Data Management Plan workshop by NTU Library. More information about the workshop is available in <https://blogs.ntu.edu.sg/ntulibrary/2016/06/29/ntu-dmp-writing-workshops-2/>

Q44 Have you attended NTU Library's Data Management Plan (DMP) workshop before?

- Yes (Skip Q45 and Q46)
- No
- Not sure

Q45 Why?*(You may select more than one option.)*

- I was not aware of the workshop
- It was not useful to my work
- It was not useful in promoting research integrity
- I had other commitments
- I was not interested in the topic
- The mode of delivery is not engaging
- Others: ________________________________________________

Q46 What would encourage you to attend the DMP workshop?  *(You may select more than one option.)*

- Certificate of completion
- Different method of delivery (e.g. webinars)
- More dates/timings
- Encouragement from PIs
- Others: ________________________________________________

Q47 How reasonable was the time needed for the DMP workshop?

- Just nice
- Too lengthy
- Too short

Q48 Currently, the DMP workshop is held monthly. How often do you think the workshop should be held?

- Once every 6 months
- Once every 3 months
- Once every 2 months
- Once a month
- Once every 2 weeks

Q49 Do you think the amount of information provided in the DMP workshop should be changed? *(You may specify what information should be included/left out.)*

- No changes needed
- Yes, more information should be provided ________________________________________________
- Yes, less information should be provided ________________________________________________

Q50 How would you rate the flow of the DMP workshop?

- Excellent
- Good
- Below average
- Poor

Q51 How effective was/were the trainer(s) in teaching the material?

- Very effective
- Moderately effective
- Slightly effective
- Not effective

Q52 How would you rate the explanations of participants' queries? Please select "not applicable" if you do not recall any queries/explanations.

- Excellent
- Good
- Below average
- Poor
- Not applicable

Q53 Was the group size helpful in facilitating discussion?

- Yes
- No, the group size should be smaller
- No, the group size should be larger

Q54 How was the quality of discussion during the DMP workshop?

- Excellent
- Good
- Below average
- Poor

Q55 How useful is the information provided in the DMP workshop to you?

- Very useful
- Moderately useful
- Slightly useful
- Not useful

Q56 How useful is the DMP workshop in promoting good research practices in your school/workplace?

- Very useful
- Moderately useful
- Slightly useful
- Not useful

Q57 How interested are you in learning more about research integrity after the DMP workshop?

- Very interested
- Moderately interested
- Slightly interested
- Not interested

Q58 How can the DMP workshop be improved? *(Optional)*

________________________________________________________________

________________________________________________________________

________________________________________________________________

________________________________________________________________

The questions in the following section is about THE COMPASS emails by Research Integrity and Ethics Office. Source: An example of an email from NTU RIEO on 6/2/2020

Q59 Have you read the monthly THE COMPASS newsletter from the Research Integrity and Ethics Office (RIEO) before?

- Yes (Skip Q60 and Q61)
- No
- Not sure

|  |
| --- |

Q60 Why? *(You may select more than one option.)*

- I do not recall receiving THE COMPASS emails from RIEO
- It was not useful to my work
- It was not useful in promoting research integrity
- I was not interested in the topic
- The mode of delivery is not engaging
- Reading the email takes too much time
- Others: ________________________________________________

Q61 What would encourage you to read RIEO's THE COMPASS emails? *(You may select more than one option.)*

- Infographics
- Less frequent
- More concise information
- Encouragement from PIs
- Others: ________________________________________________

Q62 How reasonable is the time needed to read RIEO's email?

- Just nice
- Too lengthy
- Too short

Q63 Currently, RIEO's THE COMPASS email is sent every month.
How often do you think the emails should be sent?

- Once every 3 months
- Once every 2 months
- Once a month
- Once every 2 weeks
- Once a week

Q64 Do you think the amount of information provided in RIEO's email should be changed? *(You may specify what information should be included/left out.)*

- No changes needed
- Yes, more information should be provided ________________________________________________
- Yes, less information should be provided ________________________________________________

Q65 How would you rate the flow of information provided in RIEO's emails?

- Excellent
- Good
- Below average
- Poor

Q66 How useful is the information provided in RIEO's email to you?

- Very useful
- Moderately useful
- Slightly useful
- Not useful

Q67 How useful is RIEO's email in promoting good research practices in your school/workplace?

- Very useful
- Moderately useful
- Slightly useful
- Not useful

Q68 How interested are you in learning more about research integrity after reading RIEO's email?

- Very interested
- Moderately interested
- Slightly interested
- Not interested

Q69 How can RIEO's email be improved? *(Optional)*

________________________________________________________________

________________________________________________________________

________________________________________________________________

________________________________________________________________

________________________________________________________________

End of Survey
